# Effect of surgical approach on the treatment of Morton’s neuroma: a systematic review and meta-analysis

**DOI:** 10.1186/s13047-023-00660-w

**Published:** 2023-09-07

**Authors:** Jiayao Zhang, Jing Li, Wufeng Cai, Kaiwen Zheng, Xihao Huang, Xin Rong, Qi Li

**Affiliations:** Department of Orthopedic Surgery, West China Hospital, Sichuan University, Chengdu, 610041 China

**Keywords:** Morton’s neuroma, Dorsal approach, Plantar approach, Surgery

## Abstract

**Background:**

Surgical resection of Morton’s neuroma includes dorsal and plantar approaches. However, there is no consensus on the choice of approach in clinic. The purpose of this study was to conduct a systematic review and meta-analysis to compare the surgical results of dorsal and plantar approaches.

**Methods:**

The literatures of PubMed, Cochrane library, Embase and Web of Science were searched on April 26th, 2023. A systematic review was performed using the Preferred Reporting Items for Systematic Reviews and Meta-Analysis guidelines. The data were extracted after screening the literature and evaluating the quality of the methodology included in the study. The RevMan5.4 software was used to analyze and calculate the OR value and 95% confidence interval.

**Results:**

A total of 7 randomized controlled trials and comparative studies were published, of which only 5 were included. There were 158 feet via plantar approach (plantar group, PG) and 189 via dorsal approach (dorsal group, DG). There was no significant difference between PG and DG in overall adverse events, sensory problems, incision infection and deep vein thrombosis (*p* > 0.05). In terms of scar problems, PG showed more than DG (OR, 2.90[95%CI, 1.40 to 5.98]; *p* = 0.004). Other outcome indicators such as visual analogue scale (VAS) scores and American Orthopedic Foot and Ankle Society (AOFAS) scores were difficult to be included in the comparison.

**Conclusions:**

Based on the relatively low quality and small amount of available evidence, the meta-analysis conducted produces a hypothesis that the frequency of adverse events in surgical treatment of Morton’s neuroma, dorsal approach and plantar approach may be the same, but the types are different. More high-level evidence is needed to further verify this hypothesis.

## Background

Morton’s neuroma is a common cause of forefoot pain, mainly due to repeated compression of the intermetatarsal ligament and stimulation of the plantar nerve, resulting in neural oedema, demyelination (axonal injury) and perineural fibrosis, causing local pain and discomfort during weight bearing [1–4]. According to reports, the age standardized incidence rate of Morton’s neuroma is 50.2 in males and 87.5 in females per 100,000 individuals [5]. It is a reactive degeneration of the common toe nerve, not a real neuroma, most frequently in the second or third metatarsal space [1, 6, 7]. For Morton’s neuroma patients who do not respond to conservative therapy, surgical intervention is currently regarded as the gold standard [8, 9].

In actuality, a variety of surgical techniques have been successfully employed to treat Morton’s neuroma, with neurotomy being generally used and the plantar and dorsal approaches being the most popular. Each approach provides advantages of its own, along with varying levels of patient satisfaction and complication rates. There has currently no consensus on the approach that will be most beneficial [4, 6]. The characteristics of anatomy and surgical procedures seem to be closely related to the occurrence of postoperative adverse events. Finding and removing several plantar interphalangeal nerve branches close to the level of the metatarsal head requires the dorsal approach. Attention must also be paid to minimizing the risk of damaging the dorsal cutaneous nerve branches [10]. The intermetatarsal ligament is preserved when the nerve endings are exposed adopting the plantar approach, but it also comes with a number of problems because it occupies a location in the plantar weight-bearing area [11, 12].

Reviewing the literature, Lu et al. [13] addressed several elements related to adverse events caused by the two approaches. However, they did not conduct specific comparisons of these elements and included non-comparative studies, though this might be because fewer comparative studies have been published. Making reasonable medical decisions is challenging for surgeons in the absence of adequate evidence. Importantly, the publication of new study [6] in recent years has made it possible to further compare the two approaches. Consequently, it is necessary to perform a summary analysis.

The purpose of this systematic review and meta-analysis was to compare the surgical resection of Morton’s neuroma via dorsal and plantar approaches. Accordingly, the primary postoperative objective outcome was adverse events, and the secondary was functional scores, in order to provide perspective for the selection.

## Methods

### Search strategy

Study were conducted in accordance with the guidelines of the system Review preferred reporting Project (PRISMA) [14]. Search strategies were designed using the PICOS question format: P*(Population)*: Patients diagnosed with Morton’s neuroma; I*(Intervention)*: surgical resection; C *(Comparison)*: approach; O *(Outcome)*: based on the description of postoperative adverse events; S *(Study design)*: including randomized controlled trials or other comparative studies. The literatures of PubMed, Cochrane Library, Embase and Web of Science were searched electronically on April 26, 2023, without time limit. According to the PRISMA guidelines, the retrieval strategy was executed in each database as follows: Morton* neuroma OR Morton* metatarsalgia OR interdigital neuroma. The results that met our PICOS question selection criteria were then screened.

### Research selection criteria

Inclusion criteria that met the target articles include: (a) Surgical excision of Morton’s neuromas was carried out on patients; (b) The objective was to compare the dorsal and plantar approaches, including randomized controlled trials and other types of clinical comparative studies such as cohort studies and comparative case series; (c) The patients were over the age of 18 or at least that old; (d). Research was limited to English publications. Exclusion criteria: (a) Abstracts, reports, comments, expert opinions or other incomplete published literature; (b) Republishing; (c) The data couldn’t be compared or extracted. Non-operative treatment (such as corticosteroid injection, sclerotic injection, etc.) prior to surgical intervention was not an excluded indication. The number of patients or the publication year were both unrestricted.

### Outcomes

The number of adverse events or complications reported in the articles were summarized, including postoperative sensory loss, scar-related problems, incision infection, deep vein thrombosis, etc. Furthermore, if feasible, postoperative functional scores such as visual analogue scale (VAS) scores and American Orthopedic Foot and Ankle Society (AOFAS) scores were collected.

### Data extraction

All the retrieved articles were independently screened by two researchers (JY.Z and J.L) according to the predetermined selection criteria, and the full-text qualification was evaluated after browsing the title and abstract to identify the relevant research. The differences were settled through discussion.

The two researchers independently collected the following information about each study: author, year of publication, study design, age, sex, follow-up time, sample size, surgical approach, and measurement outcomes. According to the surgical approach, the patients were divided into dorsal group (DG) and plantar group (PG).

### Evaluation of the risk of bias

The Cochrane Collaboration tool (RoB-2) [15] was used in the assessment of randomized controlled trials. The Methodological Index for Non-Randomized Studies (MINORS) [16] with adequate agreement (87.2%) [17] was employed to assess the quality and risk of bias in the included comparative studies. Those comparative studies were rated according to 8 categories: (1) a clearly stated aim, (2) inclusion of consecutive patients, (3) prospective collection of data, (4) end points appropriate to the aim of the study, (5) unbiased assessment of the study end point, (6) follow-up period appropriate to the aim of the study, (7) Loss to follow up less than 5%, and (8) prospective calculation of the study size. Each category was scored on a scale of 0 to 2, with 0 indicating high risk, 1 indicating medium risk, and 2 indicating low risk. The maximum total score was 16 points. Two independent evaluators (JY.Z and J.L) conducted the evaluation, and reached a consensus with the third (WF.C).

### Statistical analysis

The Cochrane Collaboration’s RevMan, version 5.4.1, was applied for data analysis. Heterogeneity was tested with *I*^*2*^ and the chi-squared metric. *I*^*2*^ less than 50% was considered to be within the range of acceptable heterogeneity and a fixed effect model was applied. Otherwise, the random effect model was adopted [18] *p* < 0.05 was considered to be statistically significant. Additional subgroup analysis based on category was carried out in addition to the total number of adverse events.

## Results

### Literature search findings

**Fig. 1:**
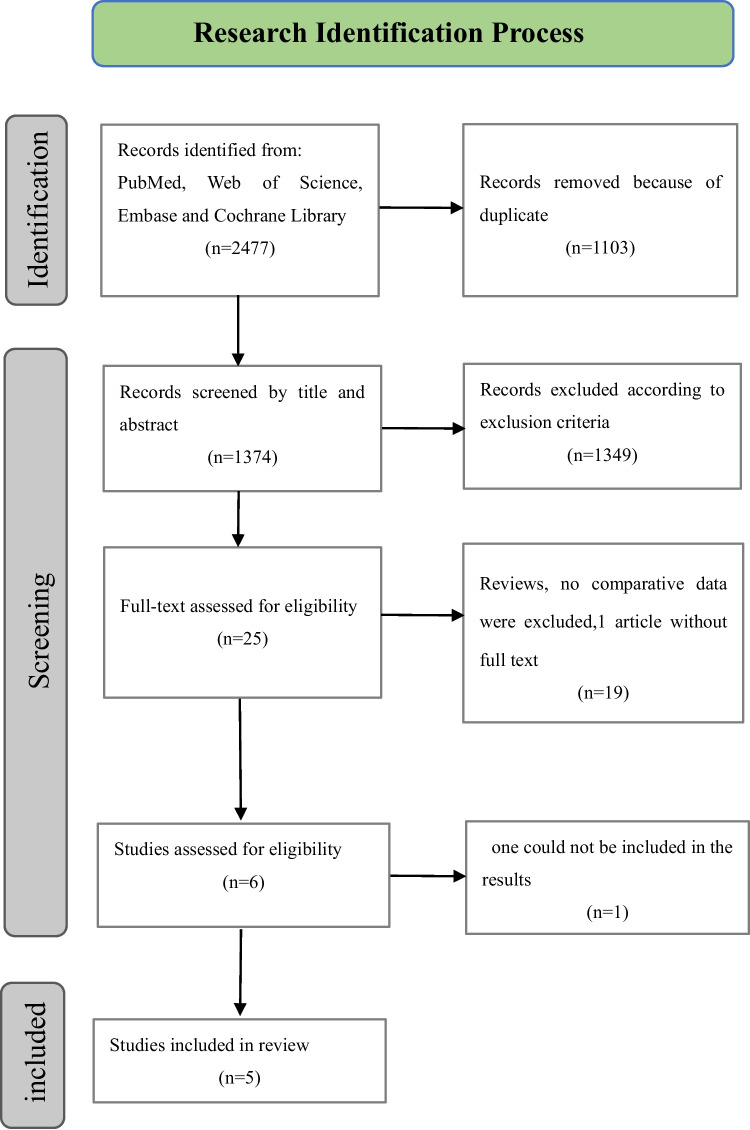
Literature selection flow diagram

According to the identified keywords, PubMed, Web of Science, Embase and Cochrane Library were searched, and a total of 2477 articles were found. After 1103 duplicated articles were excluded, the titles and abstracts of the remaining 1374 articles were screened, and 1349 articles were excluded according to the inclusion and exclusion criteria. The remaining 25 articles were further evaluated with full text. When evaluating the eligibility, one of the articles [19] that met our purpose showed a prospective comparison of the postoperative results of 55 patients treated by the two approaches, but we were unable to locate the full text despite a thorough search. The second one [20] utilized the 36-Item Short Form Health Survey (SF-36) and the Foot Function Index (FFI) for comparison, but they did not take complications into account. Five studies [6, 12, 21–23] ultimately satisfied our eligibility requirements, and the flow chart of the selected literature is shown in Fig. 1.

### Study characteristics

Four retrospective comparative studies comparing dorsal and plantar approaches to surgical treatment of Morton’s neuroma, as well as a prospective randomized controlled study, were included and reviewed (Table 1). Including the plantar approach group (*n* = 158) and the dorsal approach group (*n* = 189), a total of 347 feet of Morton’s neuroma were included.

**Table 1:** Characteristics of the included studies

**Study**	**Year**	**Design**	**Patient**	**Mean Age (years)**^**a**^		**Sex Male/Female**			**Sample size included**		**Follow-Up (months)**^**a**^		**Adverse events**^**b**^	
				PG	DG	PG		DG	PG	DG	PG	DG	PG	DG
Åkermark	2013	RCT	76	48 (23–68)	49 (25–71)	10/25		10/31	32	41	34 (28–39)	33 (28–42)	5	6
Xu	2022	RC	20	50.00 ± 13.48	46.38 ± 12.93	2/10		3/5	12	8	27.08 ± 15.34	31.5 ± 8.35	0	2
Faraj	2010	RC	36	52.08 (31–67)		2/34			20	22	18		12	6
Åkermark	2008	RC	125	52 (24–77)		41/32	24/35		73	59	29 (24–46)	37 (24–60)	4	10
Wilson	1995	RC	44	NA		3/41			21	59	at least 1 year		2	10

*Abbreviations*: *RCT* randomized controlled trial, *RC* retrospective comparative, *PG* plantar group, *DG* dorsal group

^a^mean ± SD or mean (Range); *SD* standard deviation

^b^Overall adverse events: only those explicitly stated by the author and without inclusion relationship are counted; *NA* not applicable

### Risk of bias assessment

**Fig. 2:**
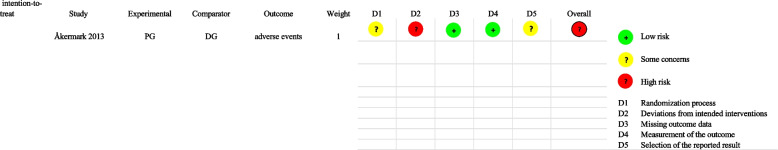
Risk of bias assessment of randomized controlled trial. *Abbreviations* PG, plantar group; DG, dorsal group

A randomized controlled trial [21] using Cochrane tool for risk assessment is shown in Fig. 2. In this study, both the subjects and the operators were not blinded. The included comparative studies were evaluated according to MINORS criteria, with an average score of 8.25 (range 7 to 10) (Table 2).

**Table 2:** Quality assessment of the non-randomized studies with the MINORS criteria

First Author	Year	Design	MINORS^a^								
			1	2	3	4	5	6	7	8	Total
Xu	2022	RC	2	1	0	1	0	2	2	0	8
Faraj	2010	RC	2	2	0	2	0	2	2	0	10
Åkermark	2008	RC	2	2	0	1	0	2	0	0	7
Wilson	1995	RC	2	1	0	1	0	2	2	0	8

*Abbreviations*: *MINORS* methodological index for non-randomized studies, *RC* retrospective comparative

^a^Only the noncomparative part of the MINORS criteria was used (ie, first 8 questions): (1) a clearly stated aim, (2) inclusion of consecutive patients, (3) prospective collection of data, (4) end points appropriate to the aim of the study, (5) unbiased assessment of the study end point, (6) follow-up period appropriate to the aim of the study, (7) Loss to follow up less than 5%, and (8) prospective calculation of the study size. Score and Risk: 0, high risk; 1, medium risk; 2, low risk. Maximum score: 16

### Outcomes of interest

**Fig. 3:**
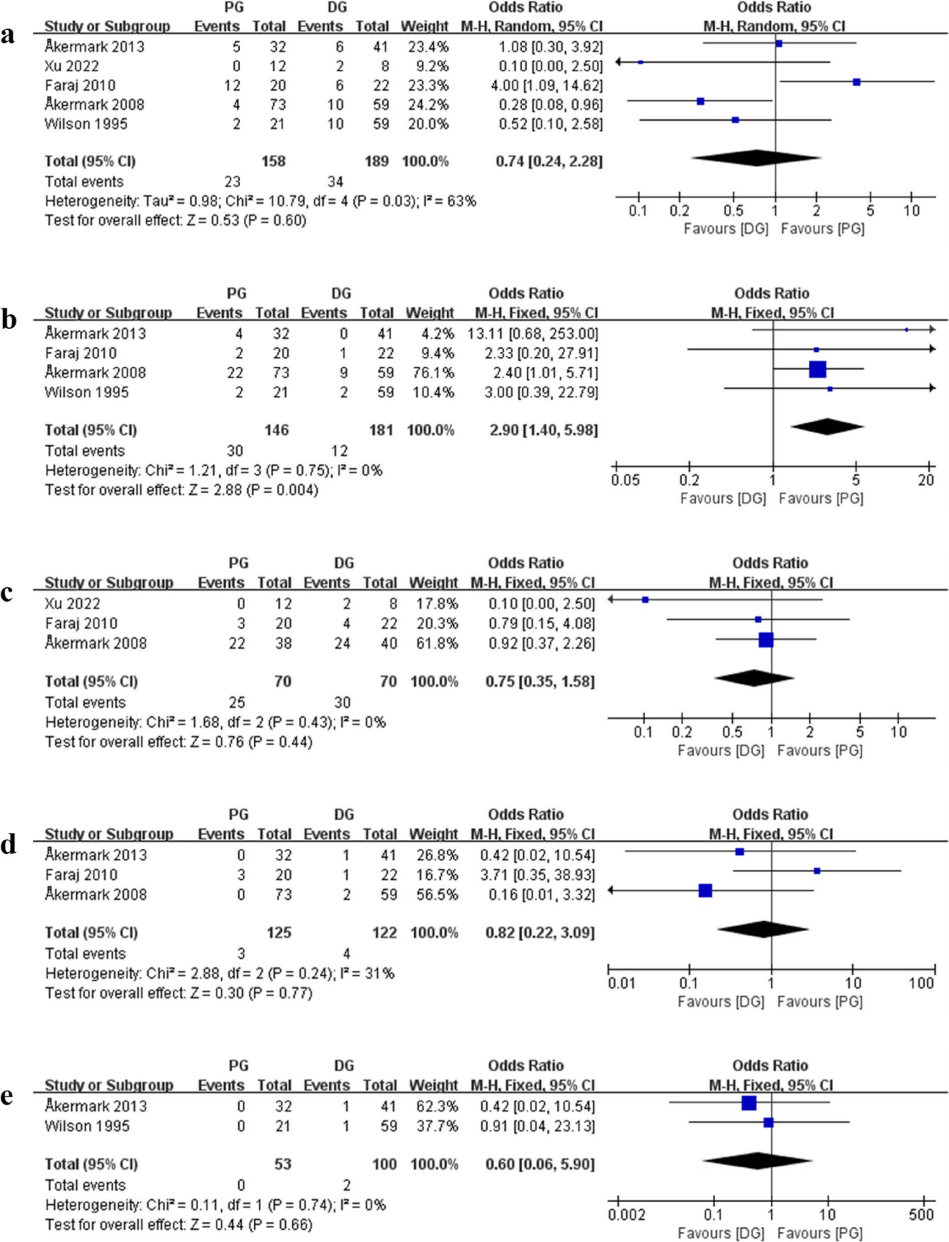
Comparison of postoperative adverse events between PG and DG. **a** overall adverse events; **b** scar-related problems; **c** sensory loss(bothersome); **d** incision infection; **e** deep vein thrombosis (DVT)

a Comparison of overall postoperative adverse events between PG and DG
  All studies recorded postoperative adverse events, including 347 feet (PG, 158 feet; DG, 189 feet), as shown in Fig. 3a. The random effect model was used to analyze the aggregate data (*p* = 0.03, *I*^*2*^ = 63%). The results showed that there was no significant difference in the occurrence of adverse events between PG and DG groups (OR, 0.74[95%CI, 0.24 to 2.28]; *p* = 0.60).
b Comparison of postoperative scar-related problems between PG and DG
  As shown in Fig. 3b, 327 feet (PG, 146 feet; DG, 181 feet) of scar-related issues (including Scar tenderness, hyperplasia, sensitivity) were identified in four study [12, 21–23]. Analysis was carried out with the fixed effect model (*p* = 0.75, *I*^*2*^ = 0%). The results showed that there was a statistically significant difference in the occurrence of scar issues between PG and DG (OR, 2.90[95%CI, 1.40 to 5.98]; *p* = 0.004).
c Comparison of sensory loss(bothersome) between PG and DG
  As shown in Fig. 3c, three studies [6, 12, 22], including 140 feet (PG, 70 feet; DG, 70 feet), revealed loss of sensation, which is bothersome. It was selected to employ the fixed effect model (*p* = 0.43, *I*^*2*^ = 0%). The results showed that there was no statistically significant difference in the occurrence of sensory loss between PG and DG (OR, 0.75[95%CI, 0.35 to 1.58]; *p* = 0.44).
d Comparison of incision infection between two groups
  As seen in Fig. 3d, incision infection occurred in three studies [12, 21, 22], totaling 247 feet (PG, 125 feet; DG, 122 feet). The fixed effect model (*p* = 0.24, *I*^*2*^ = 31%) was applied. The results showed that there was no difference between the PG and DG groups in incision infection (OR, 0.82[95%CI, 0.22 to 3.09]; *p* = 0.77).
e Comparison of deep vein thrombosis (DVT) between the two groups. Two studies [21, 23] reported the deep vein thrombosis, including 153 feet (PG,53 feet; DG,100 feet), as shown in Fig. 3e. It was selected to apply the fixed effect model (*p* = 0.74, *I*^*2*^ = 0%). Results showed that there was no difference between PG and DG (OR, 0.60[95%CI, 0.06 to 5.90]; *p* = 0.66).
f In the included studies, the lack of uniformity in postoperative functional score and difficulties in data extraction resulted in challenges incorporating the secondary outcomes for evaluation.

## Discussion

Our study found that there is insufficient consistent, standardized information to provide a comparison of the two approaches. Only 7 articles conducted a comparative study of approaches, of which 2 were difficult to be included. Reviewing of the literature, common adverse events after surgically removing a Morton’s neuroma contain scar problems, sensory loss, incision infection, missed nerve, DVT, activity restriction, and reoperation due to pain or recurrence [9, 21, 24]. Based on low-quality evidence, results from the five studies we included showed that there was no difference between the dorsal approach and the plantar approach in terms of the overall number of adverse events, and that there was no difference in regards of sensory loss, incision infection, and DVT in subgroup analysis. The scar problem, however, varies (*p* = 0.004) (Fig. 3). This may produce a hypothesis that the incidence of adverse events after neurectomy of Morton’s neuroma by dorsal approach and plantar approach is similar, but the type is different.

The dorsal approach releases the intermetatarsal ligament, and the non-weight-bearing surface facilitates early rehabilitation, provides a good overview interspace, and makes it easier to find and remove neuromas [12, 25]. Applying this approach, researchers frequently focus on the issue of easy recurrence [12, 26]. In addition, the most contentious issue is sensory loss, particularly when it is bothersome. Coughlin and Pinsonneault [27] reported that of the 71 feet treated by the dorsal approach, 36 were subjectively numb and 4 were disturbing. Giannini et al. [28] also reported more numbness after surgery. Åkermark et al. [22] discovered that DG experienced more sensory loss than PG (73% vs. 53%, respectively, *p* = 0.03). DG may have received more corticosteroid injections before and after surgery, according to their analysis. In addition, 24 out of 40 feet in DG and 22 out of 38 feet in PG were bothersome after surgery. Later, Åkermark et al. [21] pointed out that there was a greater loss of sensation in DG at baseline (*p* = 0.031), but there was no significant difference in final follow-up between the two groups (*p* = 0.062). Our study did not find the difference between the two approaches, considering that sensory tests are extremely volatile, the value is uncertain [22], and sensory loss may improve over time or patients become adaptable, we aren’t convinced that sensory loss is more likely to take place with the dorsal approach.

By remaining the intermetatarsal ligament intact when performing the plantar approach, metatarsal pain brought on by forefoot opening can be avoided, and a greater appearance index can be attained [6, 11, 29]. The plantar approach, however, resulted in a longer weight-bearing time and a significantly higher occurrence of postoperative incision infection, hematoma, and scar issues [12]. The scar issue is particularly noticeable among them. In the process of treatment 55 neuromas, Nashi et al., [19] prospectively compared the two approaches. In PG, time of weight bearing and returning to work were slower after the operation, and there were 5 painful scars in PG and only 2 in DG. In a two-year prospective follow-up study, Åkermark et al. [30] recorded 32% of patients with slight to severe scar tenderness (5% severe) after plantar approach. Furthermore, patients with scar-related symptoms reported about 6.9% [31], 5.2% [32] and 7.1% [33]. Although the dorsal approach also has a scar problem, it appears from our meta-analysis that the plantar approach has a higher occurrence. This is in line with the previous view [4, 34] that plantar approach is associated with scar formation, while dorsal approach scar formation is less.

Overall, most studies have reported the good results of the two approaches. Åkermark et al. [21] reported that there was no difference in long-term efficacy between the two groups. Similarly, a systematic review [24] pointed out that the two neurotomy approaches had success rates of 88% and 89%, respectively. For the first time, Habashy et al. [20] used SF-36 and FFI to compare, and both produced satisfactory results. Xu et al. [6] obtained similar results with VAS scores, AOFAS scores, and the Foot and Ankle Ability Measure (FAAM). Unfortunately, it makes it difficult combining the variations in outcome indicators for meta-analysis.

There are several limitations of this study, including that the sample size is still small for meta-analysis. Additionally, previous studies have not clearly defined complications, such as whether inadequate resection and stump neuroma should be marked as “complication” or “failure” [24]. In our study, two studies [22, 23] marked stump neuromas as complications. We label these results as adverse events since they are regarded in the clinic as undesirable events. Furthermore, the measurements applied to assess postoperative efficacy are frequently subjective and variable. Finally, it is essential to emphasize that the inclusion of low-quality studies has compromised the feasibility of providing recommendations for clinical practice. Although the research conducted by Åkermark et al. [21] offers the highest level of evidence, there is a high risk of bias present. In the future, more high-quality randomized controlled trials should be implemented, with efforts made to minimize the occurrence of biases. Simultaneously, establishing unified postoperative evaluation criteria is indispensable for comprehensive assessments.

## Conclusions

It provides researchers a hypothesis that the frequency of adverse events in the treatment of Morton’s neuroma by dorsal and plantar approaches may be the similar, but the types are different, based on the current small, low-level evidence. To verify this hypothesis, more substantial proof is necessary.

## Data Availability

Not applicable.
